# Book review: Becoming and being a TESOL teacher educator: Research and practice

**DOI:** 10.3389/fpsyg.2022.1047404

**Published:** 2022-11-09

**Authors:** Wang Shanshan

**Affiliations:** School of Foreign Languages and Literature, Beijing Normal University, Beijing, China

**Keywords:** language teacher educator identity, language teacher educator belief, language teacher educator emotion, language teacher education, language teaching, teacher educator

As teachers of teachers and the linchpin in teacher education, language teacher educators (TEs) play a pivotal role in designing well-structured teaching English to speakers of other languages (TESOL) teacher education programs, developing innovative pedagogy, and building a nurturing learning environment aiming for effective preparation of future TESOL teachers. Given this pivotal role of TESOL TEs and the fact that they remain under-researched globally, as noted by Golombek (2017), this volume is a timely collection to help unveil the complex and dynamic nature which feature the experiences of TEs in language teacher education. Language teachers, language TEs, and language TEs in the future may find the book a valuable learning resource.

The volume comprises an introduction and two body parts containing thirteen chapters in which a body of language TEs share, reflect upon, and discuss their experiences in their respective socio-cultural and institutional contexts. The book opens with an introductory chapter by the editors Rui Yuan and Icy Lee. In the introduction part, following an overview of TESOL TEs and a discussion of the complex and challenging realities faced by them, Yuan and Lee walk the readers through the book by highlighting the major findings and contributions of the study in each chapter.

While the inner world of TESOL teachers has been explored from various perspectives, that of TESOL TEs has largely remained under-explored. Part I (Chapters 1–7) explores the inner world (motivations, beliefs, identities, emotions, etc.) of TESOL TEs. Following the research approach of self-study, each chapter has its emphasis and could benefit TEs conceptually and empirically. Luis Banegas and Pozo Beamud in Chapter 1 situate their study in Argentina, and identify the intrinsic, extrinsic, and altruistic factors that may motivate, demotivate, and keep TESOL TEs in the profession. The longitudinal change in TESOL TEs' motivation showcased in the study may facilitate readers' understanding of the teacher learning process, and may ultimately help orient and improve TESOL teacher education programs in the long run. In the following chapter, Her, Green Eneix, and De Costa collaborate to explore how the first author of the study engages in research through the lens of “reflexivity.” The study shows the way prospective and retrospective reflexivity in research can enable and empower the teacher educator in terms of motivation and identity. Chapter 3 identifies multiple tensions experienced by the author emerging from his engagement in the teaching practicum. The study also showcases how these tensions generate professional efforts, which contribute to his role as a TESOL teacher, researcher, and teacher educator. Different from the practice-oriented research for professional development as stressed by Yung's study in Chapter 3, Yan's longitudinal self-study in Chapter 4 focuses on her beliefs about the essence of educational research beliefs and the changes in these beliefs over nearly three decades' career as a teacher educator in China. Seeking to understand TESOL TEs' identities in professional relationships with others, Minh Hue Nguyen in Chapter 5 stresses the fluid and socially negotiated nature of TESOL TE's identity by accounting for the interpretation and construction of two TESOL TEs in an Australian university. Chapters 6 and 7 touch upon the reflexivity of TESOL TEs, with the former focusing on the role of emotional reflexivity in TEs' identity and pedagogy, and the latter collaborative methodological reflections upon self-study in teacher education practices (S-STEP). Specifically, Juyoung Song in Chapter 6 shows that emotion plays an important role in bridging research and practice by providing a space for TEs to make sense of their identity, professional practice, and growth. The four reflexive narratives in Chapter 7 may encourage readers to be aware of the effectiveness of S- STEP both as a research methodology overall and one responsive to the lack of scholarship on/ by TEs and the “reflexive turn” in applied linguistics.

As its title “TESOL teacher education program design and pedagogy” suggests, Part II (Chapters 8–13) mainly discuss the teaching practice and innovations of TESOL TEs in their teacher education practice. In Chapter 8, Guofang Li and Yue Bian, following a mixed-methods study approach, explore the voices of and practice of a group of TEs by looking at their experiences of preparing pre-service teachers for ELLs. The study suggests huge disparities between these TEs' English language learning-related backgrounds, expertise, as well as teacher preparation content, and practices. Also interested in TESOL TEs' cognition and practice, Khanh-Linh Tran-Dang and Minh Hue Nguyen in Chapter 9 identify various pedagogical orientations that three Vietnamese TEs hold toward Task-based language teaching (TBLT). They find an important role of TESOL TEs' previous professional experiences in shaping their pedagogical orientations, pointing to the possibility that TESOL TEs' prior experiences could be used as affordances for their teacher education practice. Chapter 10 reports a collaborative self-study, through which the authors tackle the challenges in implementing a practice-based approach in their teacher education program in Chile. The last three chapters (Chapters 11–13) all touch upon TESOL TEs' identity with a practical focus. Mark Fraser in Chapter 11 uses self-study to detail the evolvement of his teacher educator knowledge base as a result of a capstone project and the “composition of his teacher educator identity as a practitioner, researcher, and scholar” with the help of the study. María Cristina Sarasa in Chapter 12 shares how she negotiates her teacher educator identity through engagement as a professor and participant inquirer with student-teachers in an Argentinean state university. Readers could gain valuable perspectives for studies exploring identity negotiation and professional growth from this auto-ethnography. Also focusing on identity negotiation, Bedrettin Yazan in the concluding chapter uses self-study to explore the implementation of a new teacher learning activity - critical autoethnographic narrative. Through the self-study, readers can see Yanzan's reflection upon the multi-vocal self and how this self-reflection involves further identity work.

The chapters in this volume are of important value to language teachers and language TEs- researchers in the conceptual and methodological dimensions. First and conceptually, the contributors of the volume, particularly the editors have identified the challenges in defining the term “TESOL teacher educators,” which has long been a term lacking in conceptual clarification due to the (socially, linguistically, and institutionally) heterogeneous and complex nature of TESOL teacher education. The discussions around the definition of the term would surely generate more reflection and insights concerning the boundary of the important term. Notably, the editors of the book have also identified the differences in the trajectory of becoming a TESOL teacher educator across different contexts, highlighting a novel group of TESOL TEs (with doctoral degrees and limited, even little teaching experience) emerging in geographical settings like Hong Kong and mainland China due to the recent academic climate in universities competing for external resources and striving for internalization.

Second and methodologically, the inquiries in the book provide many successful cases of the use of self-study to explore the inner world and practice of language TEs. These real-life examples prove that self-study can be an effective method for teacher educator inquiries as it provides spaces and opportunities for self-introspection and self-understanding, from which reflexivity is generated and plays an essential role. It is then also a suitable tool for language TEs to learn as both prospective and retrospective reflexivity in research facilitates TEs to deepen their understanding of the field as a result of development in the awareness of the research process. To some extent, reflexivity, as reflected in those self-study inquiries, may also help TESOL TEs to accept and embrace the messiness and obstacles in research to make them part of their identity negotiation process instead of factors to disengage them from research. Readers especially TEs and future TEs could learn along with the contributors of those self-study chapters may find that self-study can be a powerful tool and method, and have more methodological openness in their future research. With the methodological inspirations of the volume, it seems highly possible that a more diverse range of approaches to, and outcomes from, self-study of teacher education practices would emerge.

With the merits and contribution of the book mentioned, it would have been of even more help to readers, TESOL TEs especially, if it had included more theoretical discussions around and insights into the topics. Paired with the wide coverage of research topics and contexts (both geographical and educational), they would have added more to the existing literature on TESOL TEs' negotiation of meaning and their professional practice, contributing to our understanding of this important but under-explored field. This is understandable though since the impetus of the book is to provide a body of empirical research cases from diverse geographic settings and cultural backgrounds (Argentina, Australia, China, the USA, and Vietnam) for TEs to reflect upon the opportunities and challenges embedded in their experiences.

Overall, the book may encourage more TESOL teacher educator - researchers to keep seeking power, perseverance, and most importantly professional development during this particular period as the COVID-19 pandemic continues to affect people's lives and cause damage to the educational world. TESOL TEs, despite the emotional, intellectual, and practical challenges, would be empowered conceptually and methodologically to rethink their daily practice and reposition themselves as a practitioner, researcher, and scholar for more effective teacher education program design and implementation.

## Author contributions

WS has made a direct and intellectual contribution to the work and got it ready for its publication.

## Conflict of interest

The author declares that the research was conducted in the absence of any commercial or financial relationships that could be construed as a potential conflict of interest.

## Publisher's note

All claims expressed in this article are solely those of the authors and do not necessarily represent those of their affiliated organizations, or those of the publisher, the editors and the reviewers. Any product that may be evaluated in this article, or claim that may be made by its manufacturer, is not guaranteed or endorsed by the publisher.
